# Integrated genetic identification and molecular dynamics simulations in a novel *LOXL3* genetic mutation associated with Stickler syndrome

**DOI:** 10.3389/fgene.2026.1813104

**Published:** 2026-07-31

**Authors:** Min Ye, Shuai Li, Rui Li, Wen Zhang, Xianglian He, Lijun Ma, Qian Li, Wenjuan Zhuang

**Affiliations:** 1 College of Basic Medicine, Ningxia Medical University, Yinchuan, Ningxia, China; 2 The First Affiliated Hospital of Dali University, Dali, Yunnan, China; 3 Ningxia Eye Hospital, People’s Hospital of Ningxia Hui Autonomous Region, Third Clinical Medical College of Ningxia Medical University, Yinchuan, Ningxia, China

**Keywords:** *LOXL3*, novel, stickler syndrome, structural stability, variant

## Abstract

**Objective:**

Stickler syndrome (SS) is a genetically and clinically heterogeneous connective tissue disorder. This study aimed to investigate the genetic etiology in a Chinese patient associated with SS and characterize the novel pathogenic variant.

**Methods:**

Whole-exome sequencing (WES) was performed on the proband, and Sanger sequencing was conducted on both the proband and all available family members for variant validation. Variants were filtered by multiple *in silico* tools and co-segregation analysis based on American College of Medical Genetics and Genomics (ACMG) Guidelines. Structural consequences were evaluated via molecular dynamics (MD) simulations. A systematic review was conducted to summarize the genotypic and phenotypic features of previously reported *LOXL3*-related SS and early-onset high myopia (eoHM) cases, including four published case reports of independent SS pedigrees and two studies on eoHM.

**Results:**

A novel compound heterozygous *LOXL3* mutation (NM_032603.5:c.1348T>A(p.Trp450Arg); NM_032603.5:c.824dup(p.Ala277Cysfs*57)) was identified in a 5-year-old female child diagnosed with SS, who exhibited eoHM, vitreous syneresis, cleft palate, foveal hypoplasia (FVH), and mild craniofacial dysmorphism. *In silico* analysis using Variant Effect Predictor (VEP) predicted both variants as deleterious. Molecular dynamics (MD) simulations revealed that the novel NM_032603.5:c.1348T>A(p.Trp450Arg) mutation significantly destabilizes LOXL3 protein structure. Specifically, this variant increases structural flexibility and solvent accessibility while reducing hydrogen bond formation, resulting in a disordered protein conformation. Furthermore, p.Trp450Arg perturbs the copper-binding pocket and impairs copper ion coordination. Both variants co-segregated with clinical phenotypes in the family. Collectively, these functional and genetic findings, combined with clinical evidence, support the two variants as likely pathogenic and pathogenic respectively following the ACMG guidelines. The literature review revealed that high myopia is the most frequent clinical feature of *LOXL3*-related disorders, followed by FVH.

**Conclusion:**

Compound heterozygous *LOXL3* variants are associated with the SS phenotype. This study broadens the recessive mutational and phenotypic spectrum of *LOXL3*-related SS, supports refined disease subclassification and also aids genetic testing and clinical diagnosis of this disorder. It underscores the critical role of the Trp450 residue in maintaining protein structural stability and provides atomic-level insights into the molecular pathogenesis of this disorder.

## 1 Introduction

Stickler syndrome (SS) is a hereditary connective tissue disorder characterized by multisystemic manifestations involving the ocular, auditory, skeletal, and orofacial systems. (Hu et al., 2025; Parke, 2002). It represents the most common genetic cause of retinal detachment in children, frequently presenting as giant retinal tears associated with poor surgical outcomes (Ang et al., 2008). Even with successful reattachment, visual loss is usually irreversible. SS is predominantly caused by defective collagen-encoding genes. Approximately 75%–80% of cases are caused by *COL2A1* variants in autosomal dominant inheritance, and most remaining cases involve *COL11A1* or *COL11A2.* Rare variants in *COL9A1*, *COL9A2*, *COL9A3*, and lysyl oxidase-like 3 (*LOXL3*) have also been described in autosomal recessive forms of the disorder recently (Boothe et al., 2020).

SS demonstrates substantial genetic and phenotypic heterogeneity, increasing missed diagnosis and misdiagnosis in clinical practice. Distinct causative genes preferentially target different connective tissue systems and drive unique clinical manifestations. Specifically, heterozygous *COL2A1* variants usually induce severe, full-spectrum multisystem SS, with dominant ocular and skeletal lesions that confer the highest risk of blinding retinal detachment and premature osteoarthropathy. In comparison, *COL11A1* variants typically manifest as milder SS characterized by predominant auditory and orofacial defects, with relatively preserved ocular structure and function. Notably, biallelic *LOXL3* variants constitute a poorly defined SS subtype (Snead et al., 2022).

*LOXL3* (OMIM: 607163) is a member of the lysyl oxidase (*LOX*) gene family. It encodes a copper-dependent amine oxidase that catalyzes the oxidative deamination of peptidyl lysine residues in collagen (predominantly types II, IV, and VIII) and elastin precursors (Eyre et al., 1984; Laurentino et al., 2019). Biallelic pathogenic variants in *LOXL3* are associated with a spectrum of autosomal recessive connective tissue disorders, ranging from isolated early-onset high myopia (eoHM) (MYP28, OMIM: 619781) to SS with multisystem involvement. Core clinical phenotypes of *LOXL3*-related SS include severe high myopia, vitreoretinal degeneration, rhegmatogenous retinal detachment, cleft palate, hearing loss, midfacial hypoplasia, and mild skeletal dysplasia, as consistently documented OMIM annotations. However, evidence supporting the causal role of *LOXL3* in SS remains limited to merely four mutations to date (NM_032603.5:c.2027G>A(p.Cys676Tyr), NM_032603.5:c.1036C>T(p.Arg346Trp), NM_032603.5:c.824dup(p.Ala277Cysfs*57) and NM_032603.5:c.1448_1449del(p.Thr483Argfs*13)) (Alzahrani et al., 2015; Chan et al., 2019; Maddirevula et al., 2020; Klejnotowska et al., 2024), and all of these variants were identified solely through sequencing-based approaches, with limited functional validation. Accordingly, the specific pathogenic mechanisms and genotype-phenotype correlations of *LOXL3*-related SS remain poorly characterized.

Here, we identified compound heterozygous pathogenic variants in the *LOXL3* in a Han Chinese family with SS, including one novel missense variant NM_032603.5:c.1348T>A(p.Trp450Arg) and one previously reported variant NM_032603.5:c.824dup(p.Ala277Cysfs*57) (Maddirevula et al., 2020). Our findings further support the association between biallelic *LOXL3* variants and the SS phenotype. To elucidate the underlying molecular etiology, we conducted molecular dynamics (MD) simulations to characterize the atomic-level structural perturbations induced by the novel variant NM_032603.5:c.1348T>A(p.Trp450Arg) in order to explore its pathogenesis. In addition, we systematically summarized the genotype and phenotype features of *LOXL3*-related SS and eoHM refine the genotype-phenotype correlations of this disease spectrum.

## 2 Materials and methods

### 2.1 Subjects and clinical examinations

A female child who was diagnosed with Stickler syndrome (SS) according to the diagnostic criteria (Rose et al., 2005) and three other available family members (parents and brother) were recruited from the Ophthalmology Department of the Ningxia Hui Autonomous Region People’s Hospital. Prior to peripheral blood collection, written informed consent was obtained from the participants’ statutory guardians. This study was approved by the Ethics Committee of the Ningxia Hui Autonomous Region People’s Hospital (No. 2022-NZR-147) and was conducted in accordance with the principles of the Declaration of Helsinki.

Both the proband and other family members underwent a detailed medical history review and comprehensive physical examinations performed by an ophthalmologist. The assessments included best-corrected visual acuity (BCVA), slit-lamp examination, dilated fundus examination, intraocular pressure (IOP) measurement, macular Optical Coherence Tomography (OCT), fundus photography, B-scan ultrasonography. Additional evaluations included examinations of maxillofacial appearance to assess the palatal and jaw, nasal morphology, and the structure of spinal bone and cartilage. The diagnosis of SS was based on the diagnostic criteria established by Rose et al. (2005). Genomic DNA was extracted from peripheral blood samples collected from the participants.

### 2.2 Whole-exome sequencing (WES) and variant filtering

WES was performed on the genomic DNA from the proband using the Illumina HiSeq 2000 platform. The detailed methodology for WES followed procedures identical to those described in our previous study (Ye et al., 2023). We subsequently filtered out variants with a global minor allele frequency (MAF) greater than 0.001 in public population databases [Exome Aggregation Consortium (ExAC), 1000 Genomes Project (1000G), and Genome Aggregation Database (gnomAD)], as well as noncoding variants that did not affect splice sites according to the ACMG guidelines (Richards et al., 2015). We further excluded synonymous variants and missense variants predicted to be benign using at least one of the following tools: Polyphen-2, SIFT, or MutationTaster. After stepwise filtering, the variants were identified and selected for further analysis.

### 2.3 Sanger sequencing and co-segregation analysis

To validate the candidate pathogenic variants identified by WES, we performed Sanger sequencing and familial co-segregation analysis in the proband and three additional available family members. Specific primers targeting the *LOXL3* gene variants were designed using Primer 3.0 software. The target fragments were amplified by polymerase chain reaction (PCR) under appropriate conditions with the designed primers. The resulting PCR products were subsequently purified and sequenced on an ABI 3030 genetic analyser (Applied Biosystems, USA).

### 2.4 Conservation analysis

Multiple sequence alignment (MSA) of amino acid sequences was performed using ClustalX 2.1 software. The orthologous protein sequences of LOXL3 across different species were retrieved from the UniProt database and NCBI RefSeq database. We performed cross-species orthologous protein multiple sequence alignment covering nine mammalian species: *Homo sapiens* (human), *Sus scrofa* (pig), *Macaca mulatta* (rhesus monkey), *Bos taurus* (cattle), *Rattus norvegicus* (rat), *Mus musculus* (mouse), *Ovis aries* (sheep), *Delphinus delphis* (common dolphin), and *Capra hircus* (goat), to evaluate the evolutionary conservation of the amino acid sites where the variants are located.

### 2.5 Structural modelling and molecular dynamics (MD) simulations

To evaluate and compare the conformational changes and stability between the wild-type and mutant proteins, we conducted 100 ns MD simulations. The initial protein structures, predicted by AlphaFold, were used as inputs for the all-atom MD simulations performed with AMBER 18 software (Salomon-Ferrer et al., 2013). Before simulations, the proteins were described using the Amber ff14SB force field (Maier et al., 2015). The LEaP module was used in each system to add hydrogen atoms to system. A truncated octahedral TIP3P solvent box was added at a distance of 10 Å from the system, and Na+/Cl-ions were added to balance the system charges. Finally, the topology and parameter files for the simulations were output. Prior to production simulations, the system energy was minimized using 2,500 steps of the steepest descent method followed by 2,500 steps of the conjugate gradient method. After minimization, the system was heated gradually from 0 K to 298.15 K over 200 ps under constant volume conditions. A 500 ps NVT (canonical ensemble) equilibration was subsequently performed at 298.15 K to ensure uniform solvent distribution. This was followed by a 500 ps NPT (isothermal–isobaric ensemble) equilibration to stabilize the system under constant pressure. Finally, a 100 ns NPT production simulations was performed for each system under periodic boundary conditions. During the simulations, the nonbonded cutoff was set to 10 Å, long-range electrostatic interactions were treated with the particle mesh Ewald (PME) method, hydrogen bonds were constrained using the SHAKE algorithm (Kräutler et al., 2015), and temperature was regulated using the Langevin algorithm with a collision frequency γ of 2 ps^-1^. The system pressure was maintained at 1 atm, the integration time step was 2 fs, and trajectory data were recorded every 10 ps for subsequent analysis. Following the simulations, the root mean square deviation (RMSD), the root mean square fluctuation (RMSF), the solvent accessible surface area (SASA), and the radius of gyration (Rg) were computed for each residue using the software’s built-in analysis tools. Additionally, three independent simulations runs with different initial velocity seeds were performed, and all quantitative results were reported as mean ± standard deviation. Student’s t-test was applied to evaluate differences between groups.

### 2.6 Systematic review methodology

To provide an overview on up-to-date contribution of genetic variants to the phenotype, we summarized relevant reports published in the last 10 years. The literature research was performed according to the Preferred Reporting Items for Systematic Reviews and Meta-Analyses (PRISMA) in the PubMed database. The following key words were used [“high myopia” OR “early-onset myopia” OR “pathologic myopia” OR “Stickler syndrome” OR “vitreoretinal degeneration” OR “Robin sequence” OR “sensorineural hearing loss” OR “spondyloepiphyseal dysplasia”] AND [“*LOXL3*” or “lysyl oxidase-like 3”]. The search results were last updated on 1 October 2025 and limited to studies published in English. A flowchart of the literature selection process was provided in Supplementary Materials (Supplementary Figure S1). The methodological quality and risk of bias of the included studies were assessed using the Joanna Briggs Institute (JBI) Critical Appraisal Checklist for Case Reports and Case Series. Studies scoring ≥6 points were considered high quality. The results of the JBI appraisal were presented in Supplementary Table S1.

## 3 Results

### 3.1 Clinical case description

**FIGURE 1:**
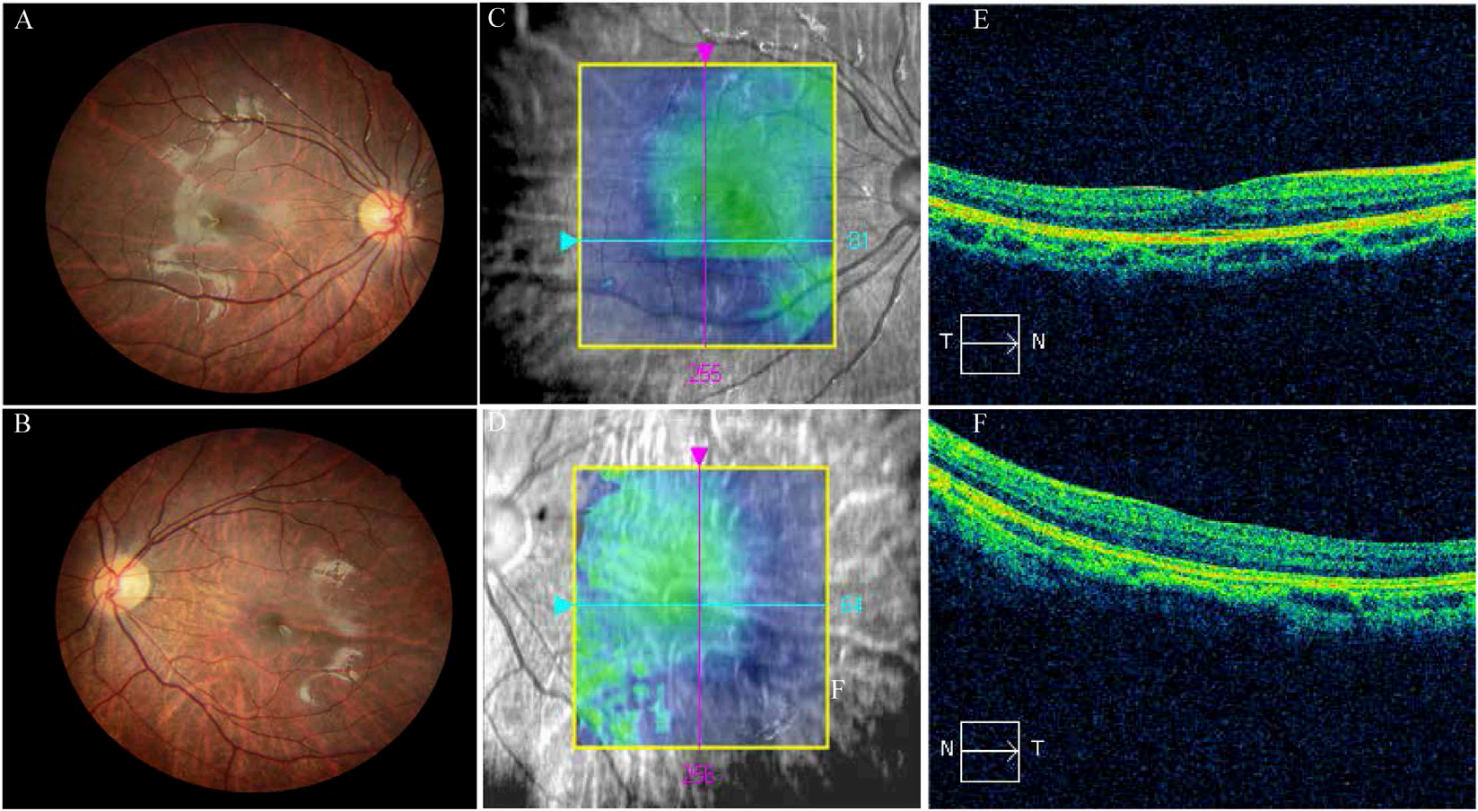
Bilateral fundus photographs and macular optical coherence tomography (OCT) of the proband. **(A,B)** Bilateral myopic fundus without prominent myopic crescent. **(C,D)** Reference infrared fundus images demonstrating the precise positioning of the OCT scan lines. **(E,F)** Bilateral macular OCT images showing foveal hypoplasia (FVH).

A 5-year-old female child with Stickler syndrome (SS) was enrolled in this study. She was born to a non-consanguineous Chinese family with no positive family history of SS, early-onset high myopia (eoHM), or related ocular/systemic manifestations of hereditary connective tissue disorders. Her initial presentation was eoHM, which was diagnosed and managed with glasses at her first ophthalmology visit. Upon further evaluation and a detailed medical history review, additional features, including vitreous anomalies, cleft palate, midface flattening and micrognathia, were identified. Ocular examination revealed bilateral visual impairment, with a best corrected visual acuity of 0.3 in both eyes. Refractive errors were significant, −13.50 D myopia with −1.25 D astigmatism in the right eye and −16.25 D myopia with −3.25 D astigmatism in the left eye. The axial lengths were elongated at 26.12 mm (right) and 26.89 mm (left). Slit-lamp examination revealed normal anterior segments in both eyes. Dilated fundus examination indicated mild bilateral vitreous liquefaction and a leopard fundus appearance without crescent peripapillary atrophy (Figures 1A,B). Her condition was classified as category 1 myopic maculopathy according to the Ohno-Matsui classification system (Ohno-Matsui et al., 2015). Macular Optical Coherence Tomography (OCT) confirmed bilateral foveal hypoplasia (FVH) (Figures 1E,F), while the corresponding foveal scanning coordinates are shown in Figures 1C,D. No abnormalities were observed in her parents and younger brother.

### 3.2 Identification of biallelic pathogenic *LOXL3* variants in the proband

**FIGURE 2:**
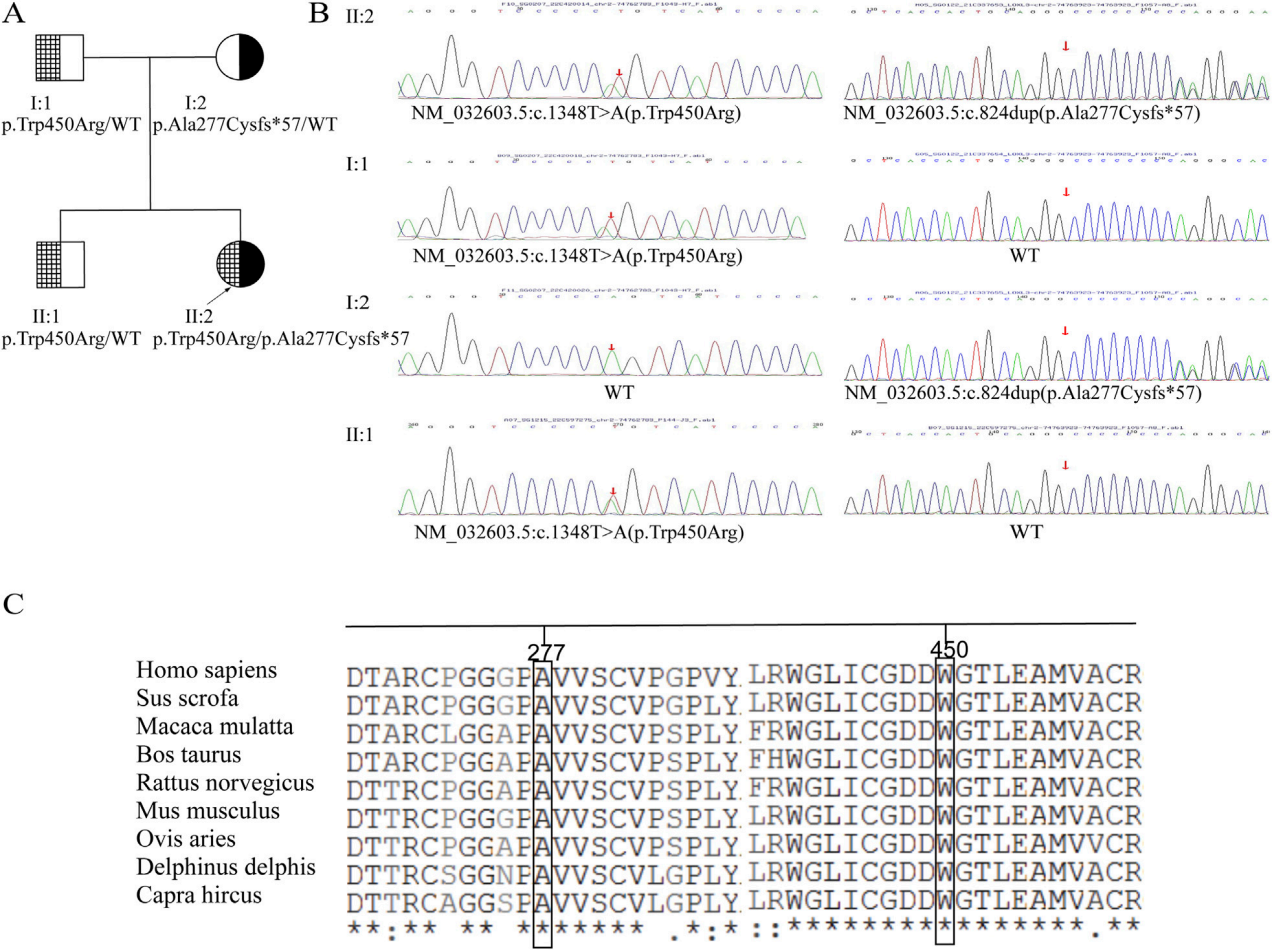
Family pedigree, Sanger sequencing validation, and evolutionary conservation analysis. **(A)** Family pedigree. Squares: males; Circles: females; WT: wild-type allele. The proband is pointed by a black arrow. A half-filled inner small squares indicates heterozygosity for mutant allele 1 (NM_032603.5:c.1348T>A(p.Trp450Arg)), and half solid black filling indicates heterozygosity for mutant allele 2 (NM_032603.5:c.824dup(p.Ala277Cysfs*57)). Compound heterozygotes are shown with combined filling. Amino acid changes are listed under each individual. **(B)** Sanger validation in the proband and family members. Red arrows indicate the location of WT and corresponding mutational sequence. **(C)** Conservation analysis of the mutated residues across nine species. Mutant sites are marked with boxes.

A compound heterozygous variant (NM_032603.5:c.1348T>A(p.Trp450Arg) and NM_032603.5:c.824dup(p.Ala277Cysfs*57)) in *LOXL3* was detected in the 5-year-old girl with SS. Both variants were confirmed by Sanger sequencing. The pedigree and sequencing data were shown in Figures 2A,B. Co-segregation analysis performed by Sanger sequencing resolved the parental origin of the two *LOXL3* variants. Specifically, the NM_032603.5:c.1348T>A(p.Trp450Arg) variant was transmitted paternally and carried by the unaffected younger brother, whereas the NM_032603.5:c.824dup(p.Ala277Cysfs*57) variant was of maternal origin and not detected in the younger brother (Figures 2A,B). Their co-segregation pattern consistent with autosomal recessive inheritance.

**FIGURE 3:**
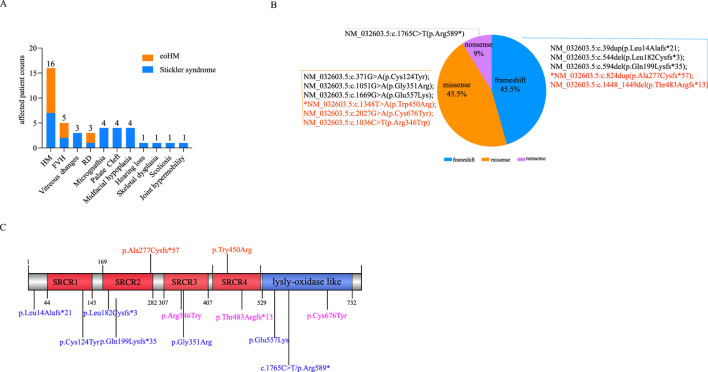
Genotype and phenotype distributions based on the current and previous studies. **(A)** Phenotype distribution of *LOXL3* variants in patients with Stickler syndrome (SS) and early-onset high myopia (eoHM).The orange area represents patients with eoHM, and the blue area represents patients with SS. The affected number was presented on each bar. HM, high myopia; FVH, foveal hypoplasia; RD, retinal detachment. **(B)** Distribution of variant types associated with SS and eoHM. SS-associated variants are colored red, and eoHM-related variants are colored black. Variants identified in this study are marked with asterisks (*). **(C)** Distribution of pathogenic and likely pathogenic variants within the LOXL3 protein in relation to SS and eoHM. Functional protein domains are denoted in red and blue. Variants uncovered in the present study are positioned above the linear protein schematic, and those documented in prior literature are positioned below. SS-implicated variants are rendered in purple (previous studies) and red (current work), whereas eoHM-associated variants are shown in blue.

The missense variant NM_032603.5:c.1348T>A(p.Trp450Arg) is novel with no prior documentation in published literature or public variant databases. It is absent in 1,000 Genomes, ExAC, and is exceedingly rare in gnomAD (frequency = 0.00000399757). *In silico* pathogenicity predictions consistently yielded deleterious results across all four assessed tools: REVEL (Damaging, score = 0.901), SIFT (Deleterious, score = 0.00), PolyPhen-2 (Probably damaging, score = 0.999) and MutationTaster (Disease-causing, score = 1) (Table 1). At the protein level, this variant results in a substitution of tryptophan with arginine position 450. Notably, the Trp450 residue is located within the scavenger receptor cysteine-rich (SRCR) domain of the LOXL3 protein (Figure 3C), a core functional domain critical for protein biological activity. Multiple sequence alignment further revealed that this tryptophan residue is highly evolutionarily conserved across mammalian species (Figure 2C), underscoring the potential deleterious impact of the amino acid substitution.

**TABLE 1:** Computational assessments of variants for the proband (II 2) with SS.

Gene	Exon	Nucleotide changes	Protein changes	Status	1000G	gnomAD	Revel	SIFT	Polyphen-2
*LOXL3*	8	NM_032603.5:c.1348T>A	p.Trp450Arg	Het	Novel	0.00000399757	D(0.901)	D(0)	PD(0.999)
*LOXL3*	5	NM_032603.5:c.824dup	p.Ala277Cysfs*57	Het	Novel	0.000192325	-	-	-

SS, stickler syndrome; 1000G, 1000 Genomes Project; gnomAD, genome aggregation database; Het, heterozygote; D damaging/deleterious; PD, probably damaging; –, not documented.

In contrast, the other variant NM_032603.5:c.824dup(p.Ala277Cysfs*57) detected in the girl has previously been reported in a sporadic case of high myopia, retinal detachment, and cataract (Maddirevula et al., 2020). This variant introduces a frameshift in exon 5, resulting in a premature termination codon. According to ACMG guidelines (Richards et al., 2015), both variants were classified as pathogenic. The frameshift variant NM_032603.5:c.824dup(p.Ala277Cysfs*57) met the criteria for PVS1, PS1, PM2, and PP3. The missense variant NM_032603.5:c.1348T>A(p.Trp450Arg) satisfied the criteria for PM1, PM2, PP3, and PP1. A comprehensive summary of variant characteristics, including population frequencies, *in silico* predictions was provided in Table 1.

### 3.3 Results of molecular dynamics (MD) simulations analysis

To further assess the structural effects of *LOXL3* variants, we performed MD simulations. As the p.Ala277Cysfs*57 mutant has been characterized previously (Maddirevula et al., 2020), our structure-based analysis focused solely on the novel p.Trp450Arg mutant.

**FIGURE 4:**
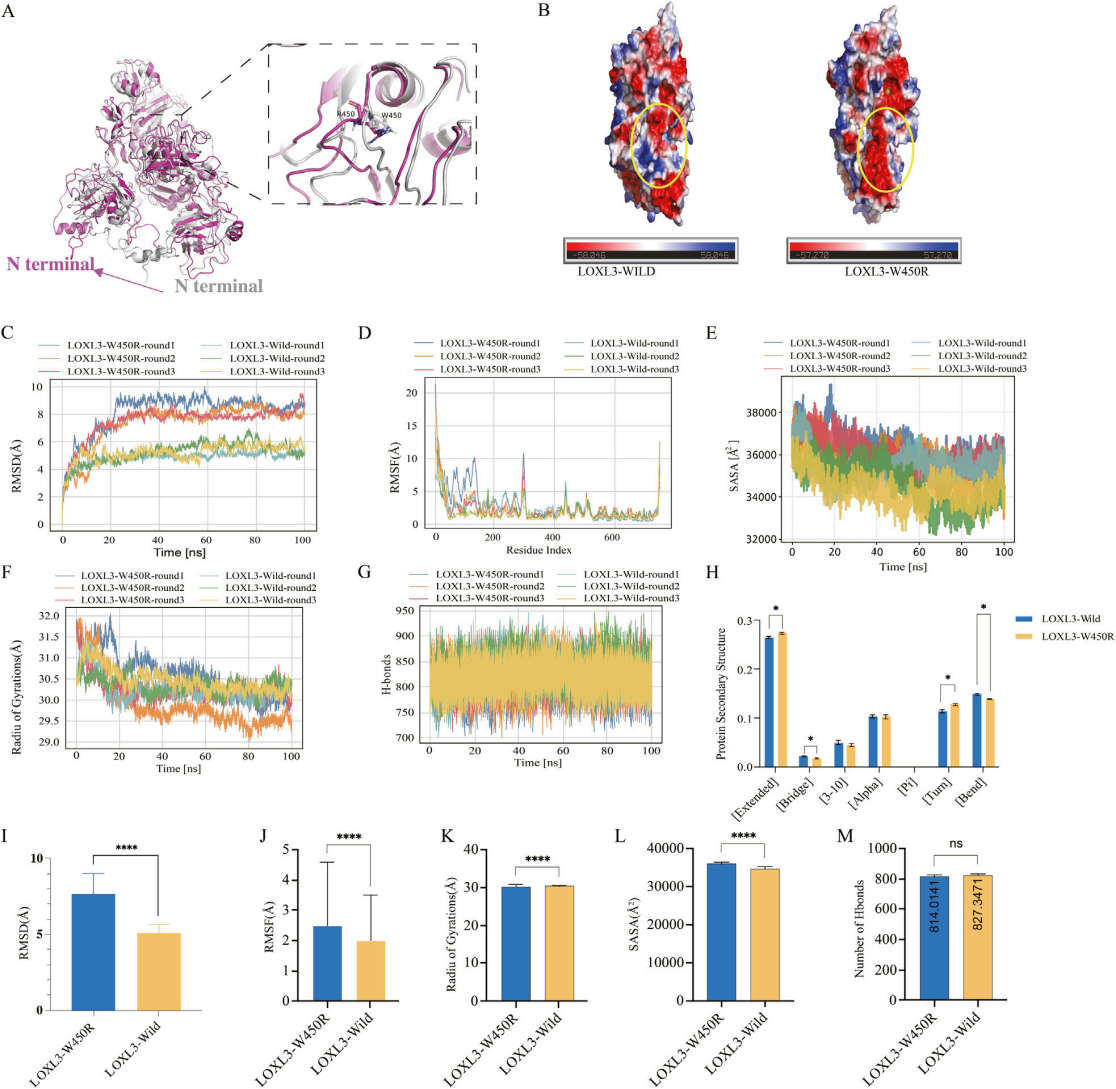
Structural models and molecular dynamics (MD) simulations results. **(A)** Superimposed wild-type (WT) (grey) and p.Trp450Arg (pink) proteins, with the variant site magnified in the top-right inset. **(B)** Electrostatic potential surfaces of WT and p.Trp450Arg LOXL3, color-coded from red (negative) to blue (positive). **(C)** Root-mean-square deviation (RMSD) profiles of WT and p.Trp450Arg proteins across the simulation trajectories. 1 Å = 0.1 nm. **(D)** Root-mean-square fluctuation (RMSF) profiles of WT and p.Trp450Arg proteins. **(E)** Solvent-accessible surface area (SASA) values of WT and p.Trp450Arg proteins. **(F)** Radius of gyration (Rg) of WT and p.Trp450Arg proteins over the simulation course. **(G)** Hydrogen bond (H-bond) counts of WT and p.Trp450Arg proteins throughout the simulation. **(H)** The secondary structure distributions of WT and p.Trp450Arg proteins. **(I–M)** Bar plots of statistical comparisons for the above metrics. Asterisks (*) indicate statistically significant differences (p < 0.05, 95% confidence interval); “ns” denotes no statistically significant difference.

Compared with the wild-type (WT) protein, p.Trp450Arg mutant induced local conformational alterations surrounding the mutation site. In particular, the N-terminal domain (residues 1–45) exhibited a positional shift and poor overlap relative to the WT, demonstrating that the p.Trp450Arg mutation disrupts the native structural architecture of LOXL3 (Figure 4A). The electrostatic potential analysis further showed that the charge changed from positive (P) to negative (N) (Figure 4B), potentially enhancing its binding affinity for positively charged nucleic acids. The root mean square deviation (RMSD) was greater in the p.Trp450Arg system, reflecting a pronounced reduction in the overall structural stability of mutant LOXL3 (Figures 4C,I). Residue-specific flexibility was assessed by using root mean square fluctuation (RMSF) analysis. In the first 300 amino acids, the p.Trp450Arg protein exhibited greater flexibility, suggesting increased conformational disorder and diminished structural rigidity (Figures 4D,J) (p < 0.05). Consistent structural destabilization was further verified by evaluating solvent accessibility, structural compactness, and secondary structure composition. The p.Trp450Arg mutant exhibited a larger solvent-accessible surface area (SASA) and radius of gyration (Rg) values, indicating greater exposure of hydrophobic regions and a less compact protein structure (Figures 4E,F,K-L). Hydrogen bonding networks showed that the p.Trp450Arg variant possessed approximately 23 fewer hydrogen bonds with increased fluctuation amplitude, indicating that the mutation disrupts intramolecular hydrogen bond networks and impairs the internal stability of LOXL3 (Figures 4G,M). Additionly, secondary structure analysis identified an increased proportion of turn structures and a decreased proportion of bend motifs in the mutant, further validating a disordered and destabilized conformational state (Figure 4H). Taken together, these multi-dimensional MD simulations results demonstrated that the p.Trp450Arg mutation destabilized LOXL3 protein structure by disrupting intramolecular hydrogen bond networks, increasing conformational flexibility, and reducing structural compactness.

**FIGURE 5:**
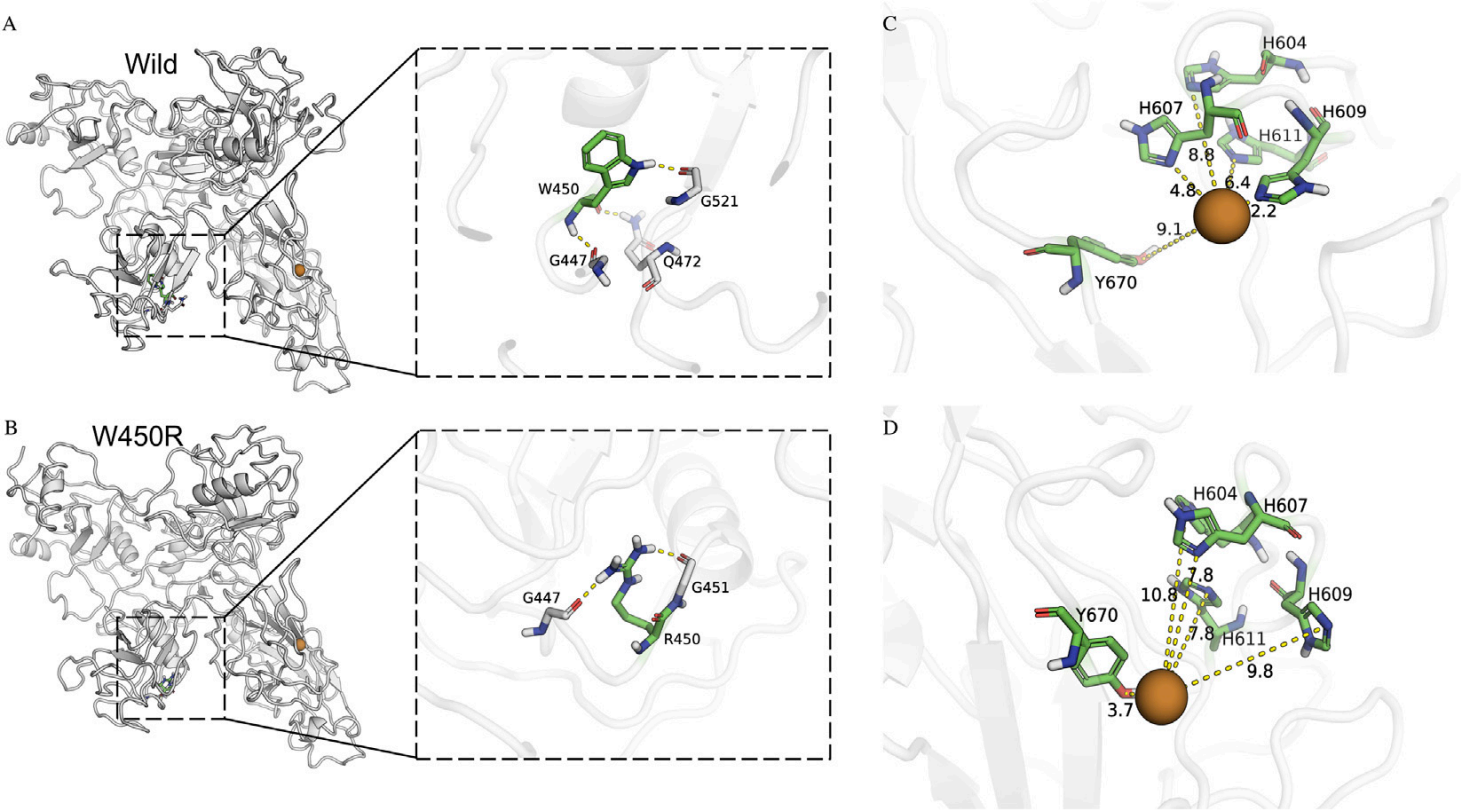
Hydrogen-bond network and side-chain conformational changes of key residues. **(A,B)** Three-dimensional (3D) structural models of wild-type (WT) and p.Trp450Arg proteins. White dashed lines denote hydrogen bonds formed between amino acid residues. **(C,D)** Side-chain conformational changes of key residues. Copper ions are depicted as brown spheres. White dashed lines indicate the distances between copper ions and their corresponding key residues.

LOXL3 exerts catalytic function via its catalytic domain, which mediates extracellular matrix cross-linking through oxidative deamination. This domain harbors a conserved copper-binding pocket formed by four key histidine residues (His604, His607, His609, and His611), which are required for copper binding and enzymatic activity. The p.Trp450Arg mutation critically disrupts side-chain interactions near the LOXL3 active site. Replacement of hydrophobic tryptophan with positively charged arginine eliminates native hydrogen bonds between Trp450 and Gln472/Gly521, disrupts local structural anchoring, and induces steric clashes (Figures 5A,B). These structural perturbations are validated by MD simulations, which show pronounced early fluctuations in Rg and SASA and reduced intramolecular hydrogen bonds (Figures 4C–G). Importantly, this pronounced conformational rearrangement propagates to the copper-binding pocket, shifting the positions of key coordinating histidine residues. Consequently, copper–histidine bond distances significantly increase, severely weakening the coordination network (Figures 5C,D). In summary, the Trp450Arg mutation destabilizes the LOXL3 catalytic domain by disrupting hydrogen bond networks and hydrophobic packing, thereby compromising copper-binding capacity and impairing LOXL3 catalytic activity.

### 3.4 Literature review of *LOXL3* genotypic and phenotypic data

To systematically characterize the genotypic and phenotypic features of *LOXL3*-related disorders, we summarized the clinical and genetic data of sixteen reported patients from fourteen unrelated families with *LOXL3*-associated Stickler syndrome (SS) or early-onset high myopia (eoHM) (Table 2). High myopia was the most prevalent core phenotype, present in all sixteen enrolled patients (100%). Notably, one 40-year-old patient had previously undergone refractive surgery, which precluded accurate assessment of his original refractive error; nevertheless, fundus examination findings were consistent with a diagnosis of high myopia. Among patients with complete refractive data, the majority presented with severe high myopia of −10.00 D or greater. The frequencies of other associated ocular and systemic manifestations were summarized in Figure 3A, with the number of affected patients indicated on each bar.

**TABLE 2:** Summary of the genotypic and phenotypic data of *LOXL3* variants.

Literature	Mutation	Phenotype											
		HM	FVH	Vitreous changes	RD	Micrognathia	Cleft Palate	Midfacial hypoplasia	Hearing loss	Skeletal dysplasia	Scoliosis	Joint hypermobility	Others
Present literature	NM_032603.5:c.824dup(p.Ala277Cysfs*57); NM_032603.5:c.1348T>A(p.Trp450Arg)	+	+	Vitreous syneresis	-	+	+	+	-	NA	NA	-	​
Alzahrani et al. (2015)	NM_032603.5:c.2027G>A(p.Cys676Tyr)	+/+	NA/NA	-/-	-/-	-/-	-/-	-/-	+/-	NA/NA	NA/NA	NA/NA	​
Li et al. (2016)	NM_032603.5:c.39dup(p.Leu14Alafs*21); NM_032603.5:c.594del(p.Gln199Lysfs*35)	+	NA	-	+	-	-	-	-	-	-	-	​
Li et al. (2016)	NM_032603.5:c.39dup(p.Leu14Alafs*21)	+	NA	-	-	-	-	-	-	-	-	-	​
Chan et al. (2019)	NM_032603.5:c.1036C>T(p.Arg346Trp)	+/myopia	NA/NA	vitreous syneresis/-	-/-	-/-	High arched/-	-/-	-/-	-/-	-/-	-/-	short stature
Maddirevula et al. (2020)	NM_032603.5:c.824dup(p.Ala277Cysfs*57)	+	NA	NA	+	-	-	-	NA	NA	NA	NA	Cataract; ID
Klejnotowska et al. (2024)	NM_032603.5:c.1448_1449del(p.Thr483Argfs*13)	+	+	Vitreous syneresis	-	+	NA	+	-	+	+	NA	Pupil dyscoria
Jiang et al. (2023)	NM_032603.5:c.39dup(p.Leu14Alafs*21); NM_032603.5:c.544del(p.Leu182Cysfs*3)	+	NA	-	-	-	-	-	-	-	-	-	​
	NM_032603.5:c.39dup(p.Leu14Alafs*21); NM_032603.5:c.1051G>A(p.Gly351Arg)	+	+	-	-	-	-	-	-	-	-	-	​
	NM_032603.5:c.39dup(p.Leu14Alafs*21); NM_032603.5:c.1669G>A(p.Glu557Lys)	+	NA	-	-	-	-	-	-	-	-	-	​
	NM_032603.5:c.371G>A(p.Cys124Tyr); NM_032603.5:c.1765C>T(p.Arg589*)	+	NA	-	+	-	-	-	-	-	-	-	​
	NM_032603.5:c.39dup(p.Leu14Alafs*21)	+	+	-	-	-	-	-	-	-	-	-	​
	NM_032603.5:c.39dup(p.Leu14Alafs*21)	+	NA	-	-	-	-	-	-	-	-	-	​
	NM_032603.5:c.39dup(p.Leu14Alafs*21)	+	+	-	-	-	-	-	-	-	-	-	​
Proportion		100%	31.3%	18.8%	18.8%	25%	25%	25%	6.3%	6.3%	6.3%	6.3%	​

HM, high myopia; FVH, foveal hypoplasia; RD, retinal detachment; NA, not available; ID, intellectual disability; +, yes; -, no.

Additionally, vitreous changes, the most characteristic changes of SS, were not highly prevalent (3/16) in this cohort. The frequency was lower than that of FVH (5/16), cleft palate (4/16), and micrognathia and midfacial hypoplasia (4/16). Moreover, in *LOXL3*-associated cases, including the present family, the observed vitreous abnormalities did not correspond to the classic membranous phenotype typically observed in SS type I or to the beaded phenotype characteristic of SS type II. Instead, they manifested mainly as vitreous syneresis or vitreous detachment (Table 2) (Figure 3A).

To date, twelve pathogenic or likely pathogenic *LOXL3* variants have been reported, comprising five frameshift, one nonsense, and six missense variants (Figure 3B). All variants except the N-terminal frameshift variant NM_032603.5:c.39dup(p.Leu14Alafs*21) localize to the SRCR or catalytic domains (Figure 3C). Allele frequency analysis revealed that NM_032603.5:c.39dup(p.Leu14Alafs*21) was the most prevalent variant (12/28 alleles), followed by NM_032603.5:c.824dup(p.Ala277Cysfs*57) (2/28 alleles), while all remaining variants were detected only once.

## 4 Discussion

In this study, we successfully identified compound heterozygous pathogenic variants in *LOXL3* via whole-exome sequencing (WES) in a Chinese child clinically diagnosed with Stickler syndrome (SS), who presented with high myopia, vitreous abnormalities, cleft palate, foveal hypoplasia (FVH), and mild craniofacial dysmorphism. The variants comprised one novel missense variant NM_032603.5:c.1348T>A(p.Trp450Arg) and one previously reported frameshift variant NM_032603.5:c.824dup(p.Ala277Cysfs*57) (Maddirevula et al., 2020). The pathogenicity of the novel variant NM_032603.5:c.1348T>A(p.Trp450Arg) was systematically evaluated via integrated bioinformatic analysis and molecular dynamics (MD) simulations. Our work presents the first atomic-level structural characterization of a *LOXL3* pathogenic variant, identifies foveal hypoplasia (FVH) as an underrecognized common ocular sign, and reveals a distinct mutational distribution across functional domains, expanding the mutational and phenotypic spectra of *LOXL3*-related SS and supporting refined disease subclassification.

In line with the core phenotypic features of SS defined by Snead et al. (Snead et al., 2022). Our systematic review confirms that congenital high myopia, vitreous dysplasia and cleft palate are shared clinical manifestations of *LOXL3*-associated autosomal recessive SS (Nixon et al., 2022). Beyond these established phenotypes, our study expands the recognized clinical spectrum of *LOXL3*-related SS, identifying additional ocular and systemic features including retinal detachment, hearing impairment, and foveal hypoplasia. High myopia remains the most prevalent phenotype with near-complete penetrance across all documented cases. Notably, the vitreous dysplasia in *LOXL3*-associated SS is distinct from the pathognomonic membranous and beaded vitreous changes characteristic of *COL2A1*-and *COL11A1*-associated SS (Soh et al., 2022). This unique vitreous signature serves as a key marker for subtype differentiation and warrants focused attention in clinical diagnostic evaluation.

Additionally, FVH emerged as the second most common clinical finding and was present in 100% of the enrolled participants who underwent macular OCT examination (5/5) (Table 2; Figure 3A). When considered across the entire cohort of sixteen patients, FVH had a prevalence of 31.3%. This is the first systematic summary of FVH in *LOXL3*-related disorders. Unlike severe, vision-impairing FVH in albinism and PAX6-related aniridia (Liu S. et al., 2021; Thomas et al., 2014), or internal retinal limiting layer persistence in *COL2A1*-related SS (Matsushita et al., 2017), *LOXL3*-associated FVH is predominantly mild (grade I/II) with uncertain effects on visual acuity. The high prevalence of FVH underscores the importance of macular OCT in the evaluation of suspected SS, and supports its potential inclusion in future diagnostic criteria. For clinical management, close monitoring of FVH progression and visual function is recommended alongside routine retinal detachment surveillance. Mechanistically, It is possible that in *LOXL3*-deficient vitreous tissue, collagen fibrils may initially form but fail to fully mature and stabilize, leading to progressive degradation and liquefaction over time rather than primary developmental failure during foveal development, although evidence for this phenomenon is lacking. We also expected that a possible link exists between FVH and the development of high myopia. Despite no direct evidence supporting this link, research has revealed that the extracellular matrix (ECM) pathway is involved in determining foveal development and that the *ACTN3* gene, which is typically associated with muscle fibre composition, is now also linked to foveal morphology (Hunt et al., 2025).

Phenotypic heterogeneity across SS subtypes is fundamentally driven by distinct pathogenic genotypes and corresponding molecular mechanisms. Unlike structural collagen gene defects that cause dominant SS, *LOXL3* encodes a lysyl oxidase that mediates collagen fiber crosslinking. Biallelic *LOXL3* deficiency impairs extracellular matrix maturation, giving rise to the distinct phenotypic profile of this recessive subtype. Our MD simulations results provide atomic-level insights into the pathogenic mechanism of the NM_032603.5:c.1348T>A(p.Trp450Arg) variant. The substitution of the highly conserved, bulky, hydrophobic tryptophan residue with a positively charged and highly hydrophilic arginine at residue 450 disrupts the local hydrophobic core of the catalytic domain, induces conformational rearrangement of the surrounding residues, and reduces overall stability of the protein. More importantly, this structural perturbation compromises the stability of the conserved histidine-copper coordination center, which is indispensable for the biogenesis of the lysyl tyrosyl quinone (LTQ) cofactor, the catalytic core of all lysyl oxidase family proteins that mediates oxidative deamination of lysine residues in collagen and elastin precursors (Zhang et al., 2018). Consequently, impaired LOXL3 enzymatic activity leads to insufficient covalent cross-linking of collagens and elastin in ECM, which is the well-established pathological basis for connective tissue defects in SS. Notably, our review of all reported *LOXL3* pathogenic variants reveals a distinct mutational distribution: nine out of twelve variants localize to the scavenger receptor cysteine-rich (SRCR) domain rather than the catalytic domain. We propose that SRCR domain variants may indirectly suppress LOXL3 catalytic activity by disrupting inter-domain interactions and compromising the structural integrity of the catalytic domain. Animal model studies have confirmed that Loxl3 is actively expressed in skeletal and cartilaginous tissues of mouse embryos and is essential for normal craniofacial and skeletal development, and loss of *LOXL3* function recapitulates human SS-like phenotypes including cleft palate, vertebral malformations, and progressive retinal degeneration (Santamaría et al., 2022; Liu Z. et al., 2021; Liu et al., 2023).

Several limitations should be acknowledged. First, our findings are based on a single family and small pooled cohort, with systemic phenotypes obtained mainly from parental reports rather than objective clinical examinations, so FVH prevalence and genotype-phenotype correlations require validation in larger multi-center cohorts. Second, while MD simulations provide robust structural evidence, direct functional validation via *in vitro* enzymatic assays or *in vivo* models is still lacking. Third, the detailed downstream pathophysiological mechanisms linking LOXL3 dysfunction to specific tissue phenotypes remain to be fully elucidated.

In conclusion, our study identifies a novel pathogenic *LOXL3* missense variant in a Chinese family with SS, provides the first atomic-level structural insights into its pathogenic mechanism, and characterizes FVH as a common underrecognized ocular feature. These findings expand the mutational and phenotypic spectra of *LOXL3*-related SS, support refined disease subclassification, and inform clinical diagnosis and counseling. Future functional studies and larger cohorts are warranted to delineate full pathogenic pathways and explore potential therapeutic targets.

## Data Availability

All data generated and analyzed in this study are available within the published article. The raw whole-exome sequencing (WES) data of the proband have been deposited in the NCBI Sequence Read Archive (SRA) database under accession number PRJNA1480185, accessible at https://www.ncbi.nlm.nih.gov/sra/PRJNA1480185.
