# Supplementary material for: Integrated genetic identification and molecular dynamics simulations in a novel LOXL3 genetic mutation associated with Stickler syndrome
Source: Front Genet. 2026 Jul 31;17:1813104. doi: 10.3389/fgene.2026.1813104 (PMC13472571; doi:10.3389/fgene.2026.1813104)
Supplement: Supplementary file 1 [file DataSheet1.pdf]

Supplementary Table 1: Assessment of case series using the Joanna Briggs Institute (JBI) Critical Appraisal Tool

Author, year	Were there clear criteria for inclusion in the case series?	Was the condition measured in a standard, reliable way for all participants included in the case series?	Were valid methods used for identification of the condition for all participants included in the case series?	Did the case series have consecutive inclusion of participants?	Did the case series have complete inclusion of participants?	Was there clear reporting of the demographics of the participants in the study?	Was there clear reporting of clinical information of the participants?	Were the outcomes or follow up results of cases clearly reported?	Was there clear reporting of the presenting site(s)/clinic(s) demographic information?	Was statistical analysis appropriate?	Total score
Alzahrani et al. 2015	Yes	Yes	Yes	Unclear	Yes	Yes	Yes	Unclear	Yes	Not applicable	7
Jiali Li et al. 2016	Yes	Yes	Yes	Unclear	Yes	Yes	Yes	Unclear	Yes	Not applicable	7
Chan, Tin K et al. 2019	Yes	Yes	Yes	Unclear	Yes	Yes	Yes	Unclear	Yes	Not applicable	7
Maddirevula et al. 2020	Yes	Yes	Yes	Unclear	Yes	Yes	Yes	Unclear	Unclear	Not applicable	6
Klejnotowska et al. 2024	Yes	Yes	Yes	Unclear	Yes	Yes	Yes	Unclear	Yes	Not applicable	7
Yi Jiang et al. 2023	Yes	Yes	Yes	Unclear	Yes	Yes	Yes	Unclear	Yes	Not applicable	7
